# What factors influence vocational medical students’ self-perceived utilization of library resources?

**DOI:** 10.5195/jmla.2026.2125

**Published:** 2026-01-01

**Authors:** Shanshan Li, Wei Jiang, Xiaoli Dai

**Affiliations:** 1 shanshanshsh@163.com, Lecturer, Jiangsu Medical College, Jiangsu, China; 2 jiangweicc233@163.com, Jiangsu Medical College, Jiangsu, China; 3 daixl_jsyy@sina.com, Associate Professor, Associate Dean, School of Medicine, Jiangsu Medical College, Jiangsu, China

**Keywords:** Library resource utilization, influencing factors, vocational medical students, regression analysis, bootstrap, space capacity, retrieval ability

## Abstract

**Background:**

Numerous studies have emphasized the crucial role of library resources in improving educational outcomes. However, there is a significant gap in research on how vocational medical students, a key group in the healthcare workforce, utilize these resources. This gap in the research highlights the need to further investigate the unique challenges and factors influencing library resource utilization in vocational medical students.

**Case Presentation:**

One hundred and seventeen vocational medical students from a medical vocational college were assessed what influenced their library resource usage. An online survey was conducted to collect data on usage patterns, satisfaction with library resources, and satisfaction with self-reported retrieval abilities. The sample included 48 males and 69 females, with an average age of 19.1±0.7 years. Of the participants, 38.5% (45 students) reported effective library resource utilization. Lasso regression and logistic regression analyses identified two key predictors: satisfaction with library's space capacity (OR 4.26, 95% CI 1.438~12.622) and satisfaction with resource retrieval ability (OR 7.362, 95% CI 1.311~41.341). ROC analysis revealed a high predictive value, with an area under the curve (AUC) of 0.866 (95% CI 0.796~0.936).

**Conclusions:**

This study identified satisfaction with library's space capacity and satisfaction with resource retrieval ability as key factors influencing library resource utilization by vocational medical students. To enhance library resource utilization, targeted strategies such as strengthening library infrastructure and improving students' information literacy should be considered.

## INTRODUCTION

Vocational medical colleges play an important role in the primary healthcare system in China. The educational model and objectives of vocational medical colleges differ significantly from those of traditional medical universities. Traditional medical universities aim to cultivate physicians equipped with fundamental clinical competencies, the ability to adapt to various medical specialties, and a strong capacity for lifelong learning [1]. In China, these programs typically span five years, and graduates are required to pass the national physician licensing examination. In contrast, vocational medical colleges offer three-year programs focused on the diagnosis and treatment of common diseases in primary care, essential clinical skills for grassroots healthcare, emergency medical techniques, and basic public health services. The curriculum emphasizes hands-on skills and vocational training, incorporating a substantial amount of laboratory work, clinical practice, and project-based learning. Upon graduation, vocational medical students take the assistant physician licensing examination and typically become assistant general practitioners or village doctors, serving primarily in community and rural healthcare institutions and providing basic health services [2]. In China, assistant general practitioners usually work in urban community health centers, while village doctors serve in rural clinics. Compared with students from traditional medical universities, vocational medical students undergo a shorter training period and generally exhibit lower levels of information literacy and lifelong learning ability [3,4,5].

Library resources are crucial for supporting medical education [6,7]. In the digital age, effectively utilizing library resources to enhance information literacy has become an important challenge for vocational medical students. Information literacy, defined as the ability to recognize when information is needed and to locate, evaluate, and use effectively the needed information, is considered a lifelong learning skill [8]. It is vital for medical students’ education and professional preparation [9,10]. In the context of vocational medical education, libraries play a distinctive role by supporting students’ acquisition of practical clinical competencies and exam-related knowledge. Library collections in vocational medical colleges often emphasize practical resources, such as guides for basic diagnostic and treatment procedures, clinical skills manuals, and materials related to the assistant physician licensing examination. Moreover, libraries may provide tailored instructional services, including training sessions on how to locate and use clinically relevant information, aiming to compensate for students’ generally lower levels of information literacy and to support the development of essential lifelong learning skills. How vocational medical students perceive and engage with these library resources may influence their ability to acquire such skills. Information literacy training is another key area supported by libraries [11,12]. By integrating information literacy instruction into medical education, medical school libraries can contribute to improving public health education and enhancing students’ information literacy and lifelong learning competencies [10,13,14].

The level of information literacy among medical students is often reflected in their use of library resources [15]. However, most existing research on library resource utilization has primarily focused on general medical students [16,17,18], often neglecting the unique characteristics and educational context of vocational medical students. This study seeks to address this gap by identifying the key factors that influence self-perceived library resource utilization and providing targeted insights into the specific needs and experiences of vocational medical students.

## CASE PRESENTATION

This survey was conducted at a vocational medical college in China that trains healthcare providers for primary care settings. The college library is a central academic resource, offering physical study spaces, print and digital materials, and access to electronic databases. The library also provides occasional training sessions to improve students’ information literacy. To better understand how vocational medical students perceive and utilize these resources, we conducted a survey focusing on their self-perceived satisfaction with various aspects of library services and facilities. The aim was to identify key factors influencing resource utilization and to inform evidence-based improvements in library support for vocational medical education.

The survey questionnaire covered the following topics: age, gender, weekly hours spent in the library (<1 hour, 1~6 hours, >6 hours); satisfaction with the library's space capacity (dissatisfied, neutral, satisfied); satisfaction with the library's physical resources (dissatisfied, neutral, satisfied); satisfaction with the library's electronic resources (dissatisfied, neutral, satisfied); satisfaction with library services (dissatisfied, neutral, satisfied); satisfaction with resource retrieval ability (dissatisfied, fair, satisfied); participation in library training or lectures (no, yes); and self-perceived library resource utilization (poor, average, good). A three-point Likert scale was used in the questionnaire to assess relevant items. The Cronbach's alpha coefficient for the questionnaire was 0.868, indicating good internal consistency. The library's space capacity refers to the availability, adequacy, and distribution of physical study spaces within the library [19,20,21]. Resource retrieval ability refers to the students’ proficiency in locating, accessing, and effectively utilizing both physical and electronic resources within the library [22].

In this study, data collection was conducted using an electronic questionnaire. The questionnaire was generated as a Quick Response (QR) code using the “Questionnaire Star” online platform. The QR code was distributed to students through class WeChat groups, inviting them to scan the code and complete the questionnaire. Students were able to access the questionnaire via smartphones or other internet-connected devices. The researchers maintained communication with class representatives to ensure timely completion of the questionnaire by the students. From May 7, 2024, to June 16, 2024, a total of 144 vocational medical students from Jiangsu Medical College were invited to participate in this study, and 117 valid responses were received, resulting in a response rate of 81.25%. The sample consisted of 48 males and 69 females, with an average age of 19.1 ± 0.7 years. The study was approved by the Ethics Committee of Jiangsu Medical College. (Approval No. 202404-PJ-002).

The case study involved constructing a lasso regression model to select variables for subsequent logistic regression analysis of self-perceived library resource utilization. The outcome variable was a binary classification of self-perceived library resource utilization (moderate or low utilization vs. high utilization). In the original questionnaire, students could select “good,” “average,” or “poor” to describe their resource utilization. However, only 5 students selected “poor,” while 67 chose “average” and 45 chose “good.” To avoid statistical instability due to the small size of the “poor” group and to enhance model interpretability, we combined the “average” and “poor” responses into a single group labeled “moderate or low utilization”. This is a statistically valid approach when dealing with sparse categories in categorical variables [23].

Nine potential independent variables were initially considered: weekly hours spent in the library, satisfaction with library's space capacity, satisfaction with the library's physical resources, satisfaction with the library's electronic resources, satisfaction with library services, satisfaction with resource retrieval ability, participation in library training or lectures, gender, and age. To choose the most relevant variables while avoiding overfitting, we used a statistical technique called lasso regression. This method helps narrow down which variables are most useful by shrinking less important ones toward zero. We used a tool called the Bayesian Information Criterion to select the best penalty level, which controls how strongly unimportant variables are reduced [24]. To make sure our results were stable, we also applied a cross-validation process. In the end, we kept only the variables that remained important after this selection process and used them in the next step of our analysis.

After selecting the important variables using lasso regression, we ran a logistic regression analysis to examine how these factors were related to self-perceived library resource utilization. To test how reliable our results were, we used a method called bootstrapping. This involves creating many new samples by randomly re-using the original data and repeating the analysis 1,000 times. This gave us more stable and trustworthy estimates of the relationships we observed.

Within the high utilization group, 51.1% were female, compared to 63.9% in the moderate or low utilization group. There was no statistically significant difference in gender distribution between the two groups. Additionally, no significant age difference was observed. However, significant differences were found regarding weekly hours spent in the library, satisfaction with the library's space capacity, satisfaction with the library's physical resources, satisfaction with the library's electronic resources, satisfaction with library services, satisfaction with resource retrieval ability, and participation in library training or lectures (Table 1).

**Table 1:** Descriptive statistics for the study variables.

Factor	Self-perceived library resource utilization		*p*-Value
	High %(n=45)	Moderate or low %(n=72)	
Gender			0.12
Male	48.9 (22/45)	36.1 (26/72)	
Female	51.1 (23/45)	63.9 (46/72)	
Age, years			0.313
(M±SD)	19.2±0.7	19.1±0.7	
Weekly library hours			0.032
<1 hour	35.6 (16/45)	51.4 (37/72)	
1-6 hours	53.3 (24/45)	47.2 (34/72)	
>6 hours	11.1 (5/45)	1.4 (1/72)	
Space capacity satisfaction			<0.001
Dissatisfied	8.9 (4/45)	29.2 (21/72)	
Neutral	11.1 (5/45)	54.2 (39/72)	
Satisfied	80 (36/45)	16.6 (12/72)	
Physical resources satisfaction			0.002
Dissatisfied	0.0 (0/45	5.5 (4/72)	
Neutral	17.8 (8/45)	43.1 (31/72)	
Satisfied	82.2 (37/45)	51.4 (37/72)	
Electronic resources satisfaction			
Dissatisfied	2.2 (1/45)	5.5 (4/72)	<0.001
Neutral	13.3 (6/45)	55.6 (40/72)	
Satisfied	84.5 (38/45)	38.9 (28/72)	
Library services satisfaction			<0.001
Dissatisfied	2.2 (1/45)	4.2 (3/72)	
Neutral	8.9 (4/45)	52.8 (38/72)	
Satisfied	88.9 (40/45)	43.0 (31/72)	
Resource retrieval ability satisfaction			<0.001
Dissatisfied	0.0 (0/45)	9.7 (7/72)	
Fair	17.8 (8/45)	65.3 (47/72)	
Satisfied	82.2 (37/45)	25 (18/72)	
Training/lecture participation			<0.001
No	44.4 (20/45)	88.9 (64/72)	
Yes	55.6 (25/45)	11.1 (8/72)	

We found that people who spend more time in the library each week were more than twice as likely to report higher self-perceived resource utilization (OR 2.707, 95% CI 1.096-6.682). Additionally, those who were more satisfied with the library's space capacity were over four times more likely to report self-perceived high resource utilization (OR 4.26, 95% CI 1.93–9.399), and those who were more satisfied with their ability to retrieve resources were over seven times more likely to report high self-perceived utilization (OR 7.362, 95% CI 2.618–20.705). Bootstrap analysis further confirmed that satisfaction with the library's space capacity (OR 4.26, 95% CI 1.438–12.622) and satisfaction with one's resource retrieval ability (OR 7.362, 95% CI 1.311–41.341) were significant factors contributing to higher perceived resource utilization (Table 2).

**Table 2:** Influencing Factors of Self-perceived Library Resource Utilization.

Factor	OR (95% CI)	*p*-Value	Bootstrap	
			OR (95% CI)	*p*-Value
Weekly library hours	2.707 (1.096~6.682)	0.031	2.707 (0.531~13.799)	0.086
Space capacity satisfaction	4.26 (1.93~9.399)	<0.001	4.26 (1.438~12.622)	0.006
Resource retrieval ability satisfaction	7.362 (2.618~20.705)	<0.001	7.362 (1.311~41.341)	0.001

**Figure 1:**
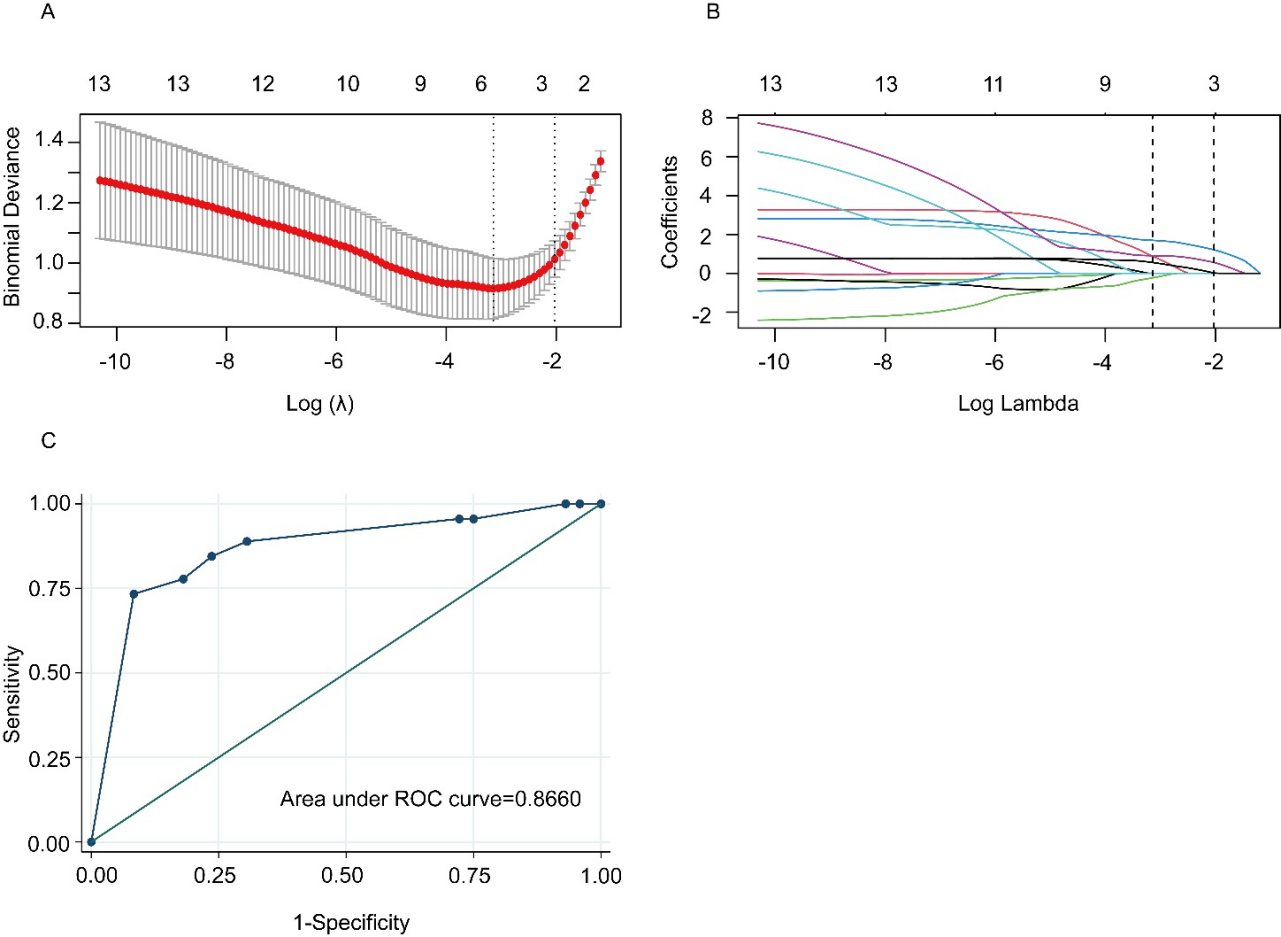
Selection of influencing factors and their predictive value (A) Identification of the optimal penalization coefficient lambda (λ) of the library resource utilization according to Bayesian information criterions. (B) A vertical line was drawn at the optimal λ value, resulting in 5 non-zero coefficients of the library resource utilization. (C) Receiver Operating Characteristic curve (ROC) for factors influencing library resource utilization, AUC = 0.866.

The reliability of these findings was confirmed using the Area Under the Curve (AUC). An AUC of 0.866 suggests that our findings are reliable and the identified factors are effective predicators of self-perceived library resource utilization (Figure 1C).

## DISCUSSION

This case report examined the self-perception of library resource utilization among vocational medical students and identified key influencing factors. Satisfaction with library space and self-perceived satisfaction with resource retrieval ability were significant predictors of self-perceived resource utilization.

In this study, the library's space capacity was identified as a significant factor influencing self-perceived library resource utilization. Prior research has demonstrated that seating availability, spatial layout, and the overall learning environment can greatly impact students’ library experience [19]. A user-centered approach that offers multifunctional spaces—such as quiet study areas and discussion rooms—is recommended to better support student needs [20,21]. Our library currently provides various physical spaces, including the main stacks, reading cafe, makerspaces, general reading rooms, electronic reading rooms, and discussion rooms. A total of 2,500 seats are available, with 788 reservable through an online system or the university's app. The library operates 91 hours per week [25].

Despite these resources, some limitations in spatial usage persist. To improve space utilization, we are considering the removal or relocation of low-use print materials to create additional space; optimization of layout to increase quiet study zones; designation of more silent areas during peak periods such as final exams; addition of small study rooms to relieve crowding; extended opening hours or more evening study space; provision of dedicated rooms for clinical case discussions and simulation during skills competition preparation; enhancement of the seat reservation system for greater accessibility; and improvement of infrastructure to ensure sufficient seating, power outlets, and access to drinking water. These improvements could enhance student satisfaction with library spaces, particularly for vocational medical students who require focused and practical learning environments.

Self-perceived resource retrieval ability was also confirmed as a key factor influencing self-perceived library resource utilization. Students who felt they had stronger retrieval skills were more likely to believe that they could effectively access and use library resources, highlighting the importance of the ability to locate required materials. This finding is consistent with previous studies [22,26,27], which indicate that limited awareness of available resources and inadequate information literacy training can hinder optimal resource use [28,29]. To improve utilization, we are considering targeted interventions such as workshops, elective courses, and personalized training sessions to enhance students’ information literacy, as well as the implementation of user-friendly retrieval systems [30].

In addition, several factors, though showing significant differences in initial analyses, were not retained in the final regression model. Weekly hours spent in the library, for example, was significantly associated with self-perceived utilization. Encouraging longer study durations and extending library access may help increase perceived resource utilization [27].

Higher satisfaction with physical and electronic resources was also linked to greater utilization. The library in this study provides a range of physical resources, including medical and general books and journals, as well as extensive electronic resources, such as SpringerLink, Wanfang Data, CNKI, EBSCO, Worldlib, and Superstar Digital Library [25]. Expanding practical resources aligned with vocational medical training–such as medical handbooks and clinical guidelines–and improving digital content (e.g., clinical case databases, telemedicine platforms) may further promote use. Similarly, library services and participation in training were associated with increased self-perceived utilization. Personalized support and retrieval guidance [27], along with orientation programs, hybrid-format workshops, and short videos, could help more students engage with library offerings. However, it should be noted that, although significant in initial analyses, these variables may not have remained significant in the final model.

Given the shorter training duration, practical orientation of vocational medical education, and the specific demands of the profession, vocational medical students have unique needs. These include greater requirements for extracurricular study time, access to practical learning spaces, and the development of information literacy to strengthen lifelong learning abilities [3,4,5]. One of the most important contributions of this study is its focus on vocational medical students, a population often overlooked in library resource utilization research. This case report offers valuable insights into vocational medical students’ perceptions of how they engage with library resources, which may help improve the impact of library offerings and ultimately quality of vocational medical education and better prepare students for their future careers.

This case report found that satisfaction with library space capacity and satisfaction with self-perceived resource retrieval ability are significant predictors of vocational medical students’ self-perceived library resource utilization. The predictive value of these factors suggests that improvements to space, layout, opening hours, and educational opportunities may enhance self-perceived resource utilization. These findings offer actionable strategies to better support the training and career development of vocational medical students.

## Data Availability

The data that support the findings of this study are available from the corresponding author for academic, non-commercial purposes upon request, provided that the request complies with institutional and ethical guidelines.

## SUPPLEMENTAL FILES

**Appendix A:** Survey Instrument
