# Supplementary material for: What factors influence vocational medical students’ self-perceived utilization of library resources?
Source: J Med Libr Assoc. 2026 Feb 17;114(1):31–7. doi: 10.5195/jmla.2026.2125 (PMC12947934; doi:10.5195/jmla.2026.2125)
Supplement: Supplementary file 1 — Appendix A: Survey Instrument [file jmla-114-1-31-s01.docx]

Survey on Library Resource Utilization

Informed consent

Dear participants, thank you for taking the time to participate in this survey. Please read the following information carefully: The purpose of this study is to understand students' use of library resources and explore related influencing factors. There is no risk in participating in this study. The possible benefit is that the library can provide students with better resources and services. This questionnaire is anonymous and does not involve personal privacy information such as name. All data will be saved anonymously. Your participation is completely voluntary and there will be no negative impact on participation or withdrawal. If you agree to participate, please continue to fill in the questionnaire. Thank you for your cooperation!

1.Your age: years old

2.Your gender:

Male

Female

3.How long do you spend in the library on average per week?

<1 hour (basically no visit)

1-6 hours

>6 hours (about 1 hour or more per day)

4.You go to the library mainly for

Self-study

Review medical professional materials (books and the Internet)

Leisure and entertainment

5.Are you satisfied with the library's space capacity? (Are there enough seats?)

Satisfied

Normal

Unsatisfied

6.Are you satisfied with the paper resources in the library? (Can they meet your needs?)

Satisfied

Normal

Unsatisfied

7.Are you satisfied with the library's electronic resources? (Can they meet your needs?)

Satisfied

Normal

Unsatisfied

8.Are you satisfied with the library's services? (Can you provide effective solutions to problems you encounter?)

Satisfied

Normal

Unsatisfied

9.Are you satisfied with your ability to retrieve resources?

Satisfied

Normal

Unsatisfied

10.Have you received any training or lectures on resource retrieval held by the library?

Yes

No

11.How do you think you utilize the school library resources?

Good

Average

Poor
